# Specificity and Plasticity of the Functional Ionome of *Brassica napus* and *Triticum aestivum* Subjected to Macronutrient Deprivation

**DOI:** 10.3389/fpls.2021.641648

**Published:** 2021-02-02

**Authors:** Galatéa Courbet, Aurélien D’Oria, Aurélia Lornac, Sylvain Diquélou, Sylvain Pluchon, Mustapha Arkoun, Anna Koprivova, Stanislav Kopriva, Philippe Etienne, Alain Ourry

**Affiliations:** ^1^UMR 950 Ecophysiologie Végétale, Agronomie et Nutritions N, C, S, INRAE, Normandie Université, UNICAEN, Caen, France; ^2^Laboratoire de Nutrition Végétale, Centre Mondial de l’Innovation Roullier Le groupe Roullier, Saint Malo, France; ^3^Botanical Institute and Cluster of Excellence on Plant Sciences (CEPLAS), University of Cologne, Cologne, Germany

**Keywords:** ionome, nutrient deficiencies, nutrient interactions, oilseed rape, sodium, vanadium, wheat

## Abstract

The composition of the functional ionome was studied in *Brassica napus* and *Triticum aestivum* with respect to the response of 20 elements under macronutrient deprivation. Analysis of relative root contents showed that some nutrients, such as Fe, Ni, Cu, Na, V, and Co, were largely sequestered in roots. After 10 days of deprivation of each one of these 6 macronutrients, plant growth was similar to control plants, and this was probably the result of remobilization from roots (Mg and Ca) or old leaves (N, P, K, S). Some tissue concentrations and net nutrient uptakes into roots were either decreased or increased, revealing multiple interactions (93 in wheat, 66 in oilseed rape) that were common to both species (48) or were species specific. While some interactions have been previously described (increased uptake of Na under K deficiency; or increased uptake of Mo and Se under S deficiency), a number of new interactions were found and some key mechanisms underlying their action have been proposed from analysis of *Arabidopsis* mutants. For example, nitrate uptake seemed to be functionally linked to Na(influx, while the uptake of vanadium was probably mediated by sulfate transporters whose expression was stimulated during S deprivation.

## Highlights

– The functional ionome is tissue and species specific and showed numerous crosstalks between nutrients in plants exposed to macronutrient deprivation. Some of the mechanisms behind this ionomic plasticity are discussed.

## Introduction

Plants require “essential” mineral elements as defined by Arnon and Stout (1939) according three rules: (i) obligatory for life cycle completion; (ii) non replaceable by other elements;, and (iii) directly involved in plant metabolism. These nutrients are usually split into two categories: macronutrients [nitrogen (N), magnesium (Mg), phosphate (P), sulfate (S), potassium (K), and calcium (Ca)] and micronutrients [boron (B), chlorine (Cl), manganese (Mn), iron (Fe), nickel (Ni), cupper (Cu), zinc (Zn), and molybdenum (Mo)], which are required in large (> 0.1% of dry weight) and low amounts (<0.1% of dry weight), respectively. Other nutrients that are non-essential, such as sodium (Na), silicon (Si), aluminum (Al), vanadate (V), cobalt (Co), and selenium (Se), can promote growth for some plant species and are also referred as “beneficial nutrients” (Marschner(eds), 2012). Plant growth and development depend on a balance between the essential mineral nutrients, which suggests the existence of numerous regulatory pathways for uptake, transport and storage in order to maintain plant requirements without excessive accumulation and the potential for toxicity (Williams and Salt, 2009; Andresen et al., 2018).

Plants, as sessile organism, have to cope with a constantly fluctuating mineral environment, and when facing macronutrient deficiencies, rely on different strategies to maintain growth and/or optimize root nutrient uptake. Indeed, Gojon et al. (2009) demonstrated that N, K, or S deficiencies in *Arabidopsis thaliana* are associated with an increase in the expression of a network of genes that modulate the quantity and activity of root transporters to improve the uptake of the missing macronutrient. However, in some instances it has been shown that a given mineral nutrient deficiency causes an induction of root-specific or non-specific transporters, and in the latter case they lead to an indirect uptake of other available nutrients. A few examples can be mentioned, such as members of the large chloride channel (CLC) family known to transport both chloride and nitrate (De Angeli et al., 2009). In the same way, crosstalk between Na^+^ and K^+^ uptake is also possible because non-selective cation uptake systems such as HKT1-type (high affinity potassium transporter), LCT1 (low-affinity cation transporter), or NSC (non-selective cation channels), are able to transport both Na^+^ and K^+^ and have also been identifiedin halophyte root cells (Schachtman and Liu, 1999). Sulfate (SO_4_^2–^), molybdate (MoO_4_^2–^), and selenate (SeO_4_^2–^) have also been considered as competitive anions for sulfate transporters (Shinmachi et al., 2010; Schiavon et al., 2012). In *Brassica napus (B. napus)*, the up-regulation of genes encoding root sulfate transporters (*Sultr1.1* and *Sultr1.2*) in S-deficient plants enhances the uptake of MoO_4_^2–^ and SeO_4_^2–^ and leads to an increase in the content of these nutrient in plant tissues (Maillard et al., 2016b). Recently, a broader description of the nutrient interactions occurring during S deficiency (Courbet et al., 2019) has highlighted other crosstalks such as between S and Fe in dinitrogen fixing species. Other examples, concerned S and Fe both required for Fe-S cluster synthesis or S, Fe, Cu, Mo, and Zn, which are required for Mo co-factor complex synthesis pathways. Other interactions between micronutrients have been reported, for example between Fe, Cu, Mn, and Zn (Grusak et al., 1999), for which root uptake can be mediated by an iron regulated transporter 1 (IRT1), and this will be the subject of a companion paper (D’Oria et al., 2020). Nevertheless, it is interesting to note that regulation of micronutrient uptake might also affect macronutrient availability. For example, Zn deprivation in barley led to an upregulation of the high-affinity P-transporter P (HVPT1 and HVPT2) allowing an increase in P uptake (Huang et al., 2000). Altogether, these studies suggest that in plants subjected to an imbalanced nutrient environment, interactions between nutrient uptake systems could be accompanied by nutritional interactions leading to large fluctuations in their internal elemental composition.

When plants are facing reduced availability of a given nutrient, remobilization of endogenous stores of the element is also an efficient way to sustain growth rates for several days or weeks, as previously described in *B. napus* plants cultivated under N (Girondé et al., 2015) or S (Abdallah et al., 2010; Girondé et al., 2014) deficiency. Usually, remobilization occurs during the vegetative stage from source organs (such as mature leaves) to support the active growth of emerging sink tissues such as young leaves (Malagoli et al., 2005; Abdallah et al., 2010). Macronutrients such as Mg, P, S, and K are remobilized from leaves in *B. napus* and *Triticum aestivum (T. aestivum)*, the latter being regarded as a highly efficient species for remobilization of most macro elements including, surprisingly, Ca (Maillard et al., 2015). Consequently, mineral remobilization, which is the process of redistributing mineral nutrients from one tissue to another, modifies the local concentration of elements. In the short term, most nutrients can be stored as organic (in proteins for example) or mineral forms in vacuoles (acting as buffer organelles between uptake and metabolic utilization) before being transported over long distances *via* conducting vessels (Peng and Gong, 2014). Except for Ca, macronutrients are considered to be highly mobile in either the xylem or phloem vessels (Etienne et al., 2018) and this enables remobilization and translocation to actively growing tissues to counterbalance transient reductions in root uptake. The processes involved in the remobilization of a given element are highly dependent on its form of storage (organic or inorganic) in plant tissues. As an example, proteins constitute the main form of N storage in many plant species, so remobilization of N requires numerous proteases that are associated with leaf senescence (Etienne et al., 2018; James et al., 2018). In contrast, S, which is mainly stored in the vacuole as SO_4_^2–^, requires an increase in the activity of tonoplastic SO_4_^2–^ transporters for its remobilization.

Over the last decade, the spreading of new technologies (Baxter, 2009) such as inductively coupled plasma optical emission spectroscopy (ICP-OES) or mass inductively coupled mass spectrometry (ICP-MS) have allowed the simultaneous and fast quantification of the content of nearly all mineral element in plant tissues [except (i) light elements such as N, C, H, O, and (ii) Cl]. Consequently, the concept of the ionome was developed and initially defined as *“*the mineral nutrient and trace element composition of an organism” but also as a “social network of mineral nutrients, controlled by a network of gene products critical for uptake, binding, transportation, and sequestration” (Baxter et al., 2008; Salt et al., 2008). It has subsequently been suggested that the ionomic composition of plant tissues might serve as a tool to reveal plant physiological status (Baxter et al., 2008; Salt et al., 2008; Baxter, 2009; Pii et al., 2015).

To our knowledge, there are very few studies that have documented modifications of the ionome content in response to individual nutrient deficiencies in contrasting plant species. This study was conducted with hydroponically grown oilseed rape and wheat submitted to 18 unique nutrient deficiencies in order to assess ionomic modifications and map all potential mineral nutrient interactions that were detectable prior to any reductions in biomass. This work focused on macronutrient deprivation and highlights the most novel and significant ionomic modifications with reference to some possible underlying mechanisms that have been targeted with available *Arabidopsis* mutants. A companion paper (D’Oria et al., 2020) focuses on the interactions resulting from deprivation of micronutrients and beneficial nutrients, with identification of the specific ionomic signature for each mineral nutrient deprivation as a Supplementary objective.

## Materials and Methods

### Plant Material and Growth Conditions

#### Growth Conditions of Oilseed Rape and Wheat

Oilseed rape (*B. napus* cv. Trezzor) and wheat (*T. aestivum* cv. Bagou) were grown in hydroponic conditions in a greenhouse (20°C day/15°C night) at the University Caen-Normandie (France) between February–March and April–May for oilseed rape and wheat, respectively. Seeds were germinated on perlite over demineralized water for 5 days in the dark and then placed under natural light for 2 days. After appearance of the first leaf corresponding to BBCH (Biologische Bundesanstalt, Bundessortenamt, and CHemische Industrie) 11 stage, 10 seedlings were transferred to plastic tanks containing 10 L of nutrient solution with a composition based on the description of Maillard et al. (2015). In this study, the composition of the nutrient solution was adapted so as to obtain plant mineral compositions that were as close as possible to those of plants grown under field conditions (Supplementary Data SD1). To do so, the mineral compositions of *B. napus* leaves grown under hydroponic conditions (Maillard et al., 2015) were compared to the leaf mineral compositions of plants grown under field conditions (derived from the data of Sarda et al. (2013) and Maillard et al., 2016b, corresponding to 194 field plots randomly harvested in France). The nutrient solution contains: 1 mM KNO_3_, 1.25 mM Ca(NO_3_)_2,_ 0.2 mM KH_2_PO_4_, 0.4 mM MgSO_4_, 0.5 μM NaFe-EDTA, 50 μM NaFe-EDDHA, 10 μM H_3_BO_3_, 3 μM MnSO_4_, 3 μM ZnSO_4_, 0.7 μM CuSO_4_, 0.008 μM (NH_4_)_6_Mo_7_O_24_, 0.1 μM CoCl_2_, 0.15 μM NiCl_2_, 0.9 mM Si(OH)_4_, 0.5 mM CaCl_2_, 0.1 mM KCl, 0.01 μM Na_2_SeO_4_, 0.1 mM K_2_SO_4_, and 0.2 mM Na_2_SiO_3_ buffered to pH 6.8 with 0.36 mM CaCO_3_. Beneficial elements are usually required at very low levels, V and Al were not provided, assuming that the traces present in water were sufficient. This solution was continuously aerated and renewed to maintain optimal nutrition conditions whenever NO_3_^–^ depletion reached thirty percent of the initial concentration according to daily NO_3_^–^ measurements conducted with test strips (Macherey-Nagel, Düren, Germany). Overall, when plant biomass and therefore growth were maximal, the nutrient solutions were changed every 2 days and every 5 days with younger plants. Natural light was supplemented with high-pressure sodium lamps (HPS 400 Watt, Hortilux Schréder, Monster, Netherlands) to obtain a mean photosynthetically active radiation (PAR) of 350 μmol photon s^–1^ m^–2^ at the top of the canopy during a 16 h (light)/8 h (dark) photoperiod.

Five replicates of two individual control plants were randomly harvested after 24 and 18 days of growth for oilseed rape and wheat, respectively. Fresh roots and leaves were separated, weighed and kept for further analysis. This harvest performed just before application of the macronutrient deprivation corresponded to day 0 (D_0_) of the deprivation experiments. The remaining plants were then split into seven subsets. Aboveground tissues of each plant were labeled with a marker pen (on leaves and petioles for wheat and oilseed rape, respectively) in order to separate tissues that developed before (old leaf blades (OLBs) for both species and old petioles (OP) for oilseed rape) or during deficiencies (young leaf blades (YLBs) for both species and young petioles (YPs) for oilseed rape) in future harvests. Control plants (without nutrient deficiency) were cultivated with the complete nutrient solution described above. Each of the 6 other batches received a specific nutrient solution with a single element omitted: N, Mg, P, S, K, or Ca. The compositions of these nutrient solutions (containing the same amount of nutrients as the complete nutrient solution except for the omitted element) are provided in Supplementary Data SD2. Each deprived solution was buffered to pH 6.8 with a specific CaCO_3_ concentration (Supplementary Data SD2) except for Ca-deprived solution where CaCO_3_ was replaced with a 1 mM KOH solution. Control and deprived plants were harvested after 10 days (D_10_). This duration of macronutrient deprivation was chosen in order to harvest the plants before any significant decline in growth and from preliminary experiments and according to Maillard et al. (2015) and Sorin et al. (2015). These studies, using oilseed rape grown under non-limiting nutrient conditions (i.e., relatively similar conditions to those used in this study), showed that plant growth was significantly reduced only after 14 days of S deprivation (Sorin et al., 2015), after 11 days of N deprivation and after 15 days of Mg, P, S, or K deprivations (Maillard et al., 2015). The maintenance of growth for more than 10 days of mineral nutrient deprivation was then explained by the ability of plant to mobilize internal mineral nutrient stores (that were plentiful as plants were previously grown under non-limiting supply) to sustain growth for such a relatively long period. Five replicates made up of two plants were randomly collected from the two available tanks and separated into old leaves (or old leaves and old petioles for oilseed rape), young leaves (or young leaves and young petioles for oilseed rape) and roots. The fresh weight of each tissue was determined and then separated in two homogenous aliquots. Then the samples were oven dried for 72 h at 70°C for dry weight determination and used for further elemental concentration quantification.

#### Growth of *Arabidopsis* Lines

*Arabidopsis* Columbia wild type (Col) and *sultr1.1* (SALK_093256) and *sultr1.2* (sel1-8; Shibagaki et al., 2002) mutants previously grown under high or low S conditions by Maillard et al. (2016b) were used for determination of their leaf V concentration. Briefly, Arabidopsis (Col or mutant lines) was grown for 17 days on agarose plates with a modified Long Ashton nutrient solution containing a high (0.75 mM) or low (0.075 mM) supply of sulfate. Plants were cultivated in a controlled environment room under a long day (16 h light/8 h dark cycle) at a constant temperature of 22°C, 60% relative humidity and a light intensity of 120 μE. s^–1^. m^–2^. Leaves were harvested, weighed, dried and ground for determination of their elemental composition.

### Element Analysis by Mass Spectrometry and Calculations

Before element analysis, all dried samples were ground to a fine powder with 0.4 mm diameter inox beads using an oscillating grinder (Mixer Mill MM400, Retsch, Haan, Germany). For total N, 1.5 mg of fine powder were placed in tin capsules before analysis with an isotope-ratio mass spectrometer (IRMS, Isoprime, GV Instruments, Manchester, United Kindgdom) linked to a C/N/S analyzer (EA3000, Euro Vector, Milan, Italy). Analysis of Cl, Si and Al was performed using a X-Ray-Fluorescence Spectrometer (XEPOS, Ametek, Berwyn, United States) from about 1 g of dry weight (DW). Cl, Si, and Al concentrations of plant samples were determined by reference to calibration curves established from international standards with known concentrations of Cl, Si, and Al.

All the remaining elements (Mg, P, S, K, Ca, B, Mn, Fe, Ni, Cu, Zn, Mo, Na, Co, V, Se) were analyzed by high-resolution inductively coupled plasma mass spectrometry (HR-ICP-MS, Element 2^TM^, Thermo Fisher Scientific, Bremen, Germany) as described by Maillard et al. (2016a). Briefly, 40 mg of dry powder were subjected to microwave acid sample digestion (Multiwave ECO, Anton Paar, les Ulis, France) with 1 mL of concentrated HNO_3_, 250 μL of H_2_O_2_, 900 μL of ultrapure water and 10 μL of internal standard solutions of gallium and rhodium (10 and 2 μg L^–1^, respectively). Thereafter, digested samples were diluted to 50 mL with ultrapure water to obtain a 2.0% (v/v) solution of nitric acid. Finally, this solution was filtered with a 0.45 μm Teflon filter. Quantification of each element was performed from calibration curves after being corrected from the recovery rate by subtracting the blank and using internal standards (Ga and Rh). In addition, the quality of mineralization and quantification was evaluated using certified reference plant material (*Citrus* leaves, CRM NCS ZC73018, Skylab, Metz, France).

The quantity of each element (Q) in a tissue (i)r at each harvest time (t) was calculated as follows:

(1) QEitDWit

Where E is the elemental concentration (ppm) and DW the dry weight (g).

Net uptake (NU) was calculated as the difference in the whole plant quantity (*i.*e., sum of all tissue quantities) between D_0_ and D_10_ as follows:

(2) NU∑i1nQiD10orD22-∑i1nQiD0

With *n* = 5 or *n* = 3 tissues for oilseed rape and wheat, respectively.

In this study and for each mineral nutrient, some results were expressed relative to control plant values as the following ratios for element concentration (E_deprived_/E_control_) or net uptake (NU_deprived_/NU_control_), while relative root nutrient content was calculated as the ratio of the root content/total plant content.

### Statistical Analysis

Data were analyzed based on an experimental design that contained five independent replicates, each consisting of a pool of two individual plants, except at D_0_ where four plants were needed to ensure enough material was available for analysis. The quantity (Q) (μg or mg) and elemental concentration (ppm) are given as the mean ± standard error (S.E) for *n* = 5. The NU corresponding to the difference in the whole plant quantity of each element between D_0_ and D_10_ was calculated by considering all random combinations between two sets of 5 replicates and thus is given as the mean ± S.E. for *n* = 25.

Statistical analyses were performed using R software (version 3.5.1) (R Core Team, 2019) and RStudio (version 1.1.456) (RStudio Team, 2020). Significant difference in the means between control and deprived plants were determined using Student’s *T*-test and differences among all treatments were established using one-way ANOVA and Tukey *post-hoc* tests.

Heatmap representations were generated using the heatmap.2 package and represent a color gradient for values relative to control content between 0.2 (red) and 5 (green). Blank cells in heatmaps correspond to non-significant variations in content or net uptake compared to control plants.

## Results

### Biomass Production During 10 Days of Macronutrient Deprivation

**FIGURE 1:**
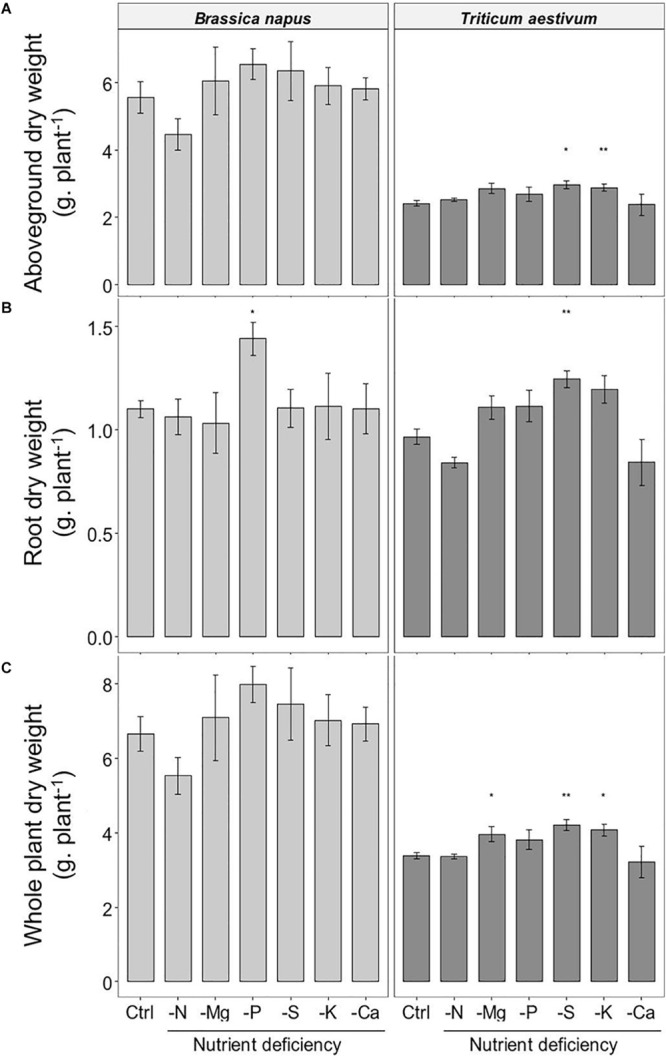
Accumulated dry weight **(A)** aboveground; **(B)** roots; **(C)** whole plant) of *B. napus* and *T. aestivum* plants, 10 days after macronutrient deprivation under hydroponic conditions. Data are given as the mean ± S.E. (*n* = 5) and significant differences between control and macronutrient-deprived plants are indicated as follows: *p < 0.05, **p < 0.01.

Compared to control plants, very few macronutrient deprivations significantly affected the biomass of the plants (Figure 1). It was only the root biomass in oilseed rape (Figure 1B) that increased significantly under the -P treatment (1.44 ± 0.08 g.plant^–1^ and 1.10 ± 0.04 for -P and control plants, respectively). In wheat, very little variation was observed in the whole biomass of the control plants (3.38 ± 0.09 g.plant^–1^, i.e., CV = 5.8%) (Figure 1C), the -S and -K treatments significantly increased the aboveground by about 20% while only S deprivation increased significantly root biomass (Figures 1A,B). For most treatments, the increase in the individual tissue biomass did not lead to a significant increase in the biomass of the whole plant, except in wheat where the -S, -K, and -Mg treatments increased the total biomass relative to control (4.21 ± 0.15, 4.07 ± 0.16, 3.96 ± 0.21, and 3.38 ± 0.09 g.plant^–1^ for −S, −K, −Mg, and control plants, respectively). Overall, the results of biomass production in both species showed that 10 day macronutrient deprivation had a moderate effect or no effect on growth.

### Species-Specific Ionome

The ionomic compositions corresponding to the elemental content of control plants in each tissue harvested at D_10_ for oilseed rape and wheat are presented in Table 1. For all tissues, the mineral nutrient content in oilseed rape was higher than in wheat, except for Si, which was found at much higher concentrations in wheat shoots.

**TABLE 1:** Mineral nutrient concentrations in parts per million (ppm), of *B. napus* and *T. aestivum* control plants at time D_10_ grown under hydroponic conditions.

		*Brassica napus*					*Triticum aestivum*		
		YLBs	OLBs	YPs	OPs	Roots	YLBs	OLBs	Roots
Macronutrients	**N**	61,030 a	43,116 b	31,843 d	29,222 d	29,598 d	42,445 b	38,569 b	31,461 d
	**Mg**	2,723 d	6,422 a	3,350 c	4,669 b	3,693 c	1,683 e	3,340 c	4,255 b
	**P**	9,382 a	5,465 c	7,647 b	5,656 c	7,454 b	5,492 c	3,440 d	4,167 d
	**S**	9,972 b	21,142 a	7,486 c	6,890 c	10,178 b	4,481 d	6,360 c	2,748 e
	**K**	47,222 b	55,803 b	91,073 a	101,813 a	52,682 b	50,639 b	50,798 b	30,241 c
	**Ca**	11,692 d	50,763 a	17,257 c	38,986 b	8,254 e	3,871 f	12,003 d	6,574 e
Micronutrients	**B**	43 b	84 a	34 c	37 c	24 d	7 e	8 e	7 e
	**Cl**	4,655 f	16,764 c	19,760 b	31,554 a	12,910 d	14,870 cd	13,834 d	10,467 e
	**Mn**	81 de	210 b	51 e	88 de	139 c	84 de	265 a	101 d
	**Fe**	162 d	163 d	77 d	63 d	905 a	167 d	396 c	578 b
	**Ni**	0.4 c	0.7 c	0.4 c	0.2 c	5.1 a	0.3 c	0.9 c	3.8 b
	**Cu**	8 cd	8 cd	4 de	3 e	26 b	11 c	12 c	47 a
	**Zn**	70 b	41 c	50 c	40 c	93 a	88 a	87 a	73 b
	**Mo**	3.0 d	6.6 a	2.0 e	3.7 c	3.2 d	2.0 e	5.9 b	1.2 f
Beneficial nutrients	**Na**	717 de	1,336 de	2,003 d	3,509 c	10,356 a	187 e	219 e	6,175 b
	**Al**	399 b	681 a	730 a	798 a	705 a	431 b	432 b	360 b
	**Si**	729 c	1,018 c	654 c	737 c	2,391 c	5,597 b	39,124 a	2,275 c
	**V**	0.05 c	0.06 c	0.04 c	0.05 c	0.24 a	0.03 c	0.16 b	0.20 ab
	**Co**	0.15 c	0.10 c	0.10 c	0.09 c	6.91 a	0.03 c	0.11 c	3.91 b
	**Se**	0.63 c	1.44 a	0.80 b	0.84 b	0.77 b	0.37 d	0.28 d	0.34 d

*Tissues developed before or after D_0_ are indicated as “young” or “old” as follows: young leaf blades (YLBs), old leaf blades (OLBs), young petioles (YPs) and old petioles (OPs). Data are given as the mean ± SE (n = 5), with each replicate (n = 5) corresponding to a pool of two individual plants, and are expressed in ppm. For each mineral nutrient, means sharing the same letter were not significantly different (Tukey post-hoc test at α = 0.05).*

In OLBs and YLBs, the K, Mn, Fe, Ni, Cu, V, and Co concentrations were mostly within the same order of magnitude for both species while the N, Mg, P, S, Ca, B, Mo, Na, Al, and Se concentrations were significantly higher in oilseed rape. In contrast, the leaf blades of oilseed rape contained much less Si, Zn, and Cl (except for the latter in YLBs) than in wheat and showed lower concentrations for some metal elements (Mn, Fe, Zn) in OLBs. In roots, except for N and Cu, all element concentrations were higher in oilseed rape. A clear age effect associated with leaf blades emerged for both species, as lower concentrations of all elements were found in YLBs compared to OLBs, except for N, P, and Zn. In roots, Fe, Ni, Cu, Na, V, Co, and Se were in much higher concentrations than in the aboveground tissues, irrespective of the species.

### Relative Root Nutrient Amounts Under Macronutrient Deprivation

**FIGURE 2:**
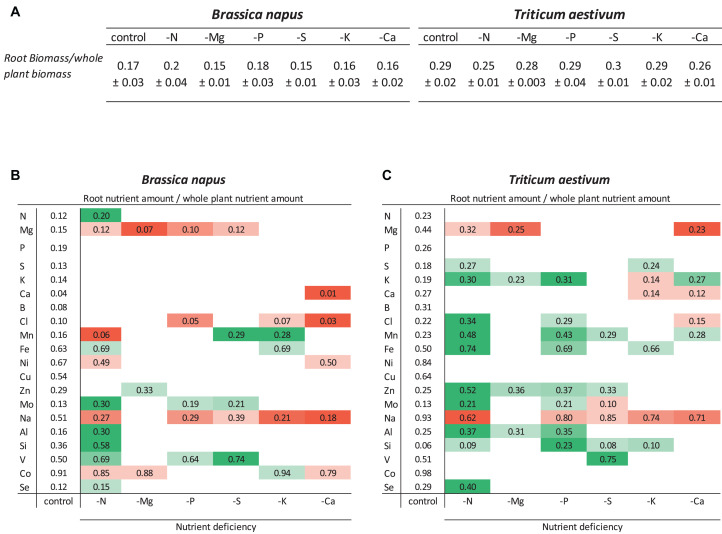
Ratio of roots to whole plant biomass **(A)** and relative root nutrient contents of *B. napus* **(B)** and *T. aestivum* **(C)** plants after 10 days of macronutrient deprivation calculated as the ratio of nutrient content in roots/entire plant. Increases (green) or decreases (red) in relative root contents are only given if they were different from control plants for *p* < 0.01. The root biomass/total plant biomass ratios are also given as the mean ± S.E. (*n* = 5) for comparisons with relative root nutrient contents.

Relative root nutrient amounts, resulting from both root nutrient uptake and shoot nutrient translocation, were calculated as the ratio of the root nutrient amount to the whole plant nutrient amount (Figures 2B,C). For an optimal interpretation, this relative root nutrient amount needs to be compared to the root biomass/whole plant biomass ratio (Figure 2). In both species, the biomass ratios of nutrient-deprived plants were similar to control plants (around 0.17 ± 0.018 and 0.28 ± 0.018 in oilseed rape and wheat, respectively), except for N deprivation, which in wheat resulted in a significantly lower biomass ratio of 0.25 ± 0.009 (*p* < 0.05) (Figure 2A).

In control plants of both species, the comparison of these biomass ratios with relative root nutrient content allowed classification of the nutrients into different categories. The first category corresponded to nutrients accumulated or sequestered in roots such as Fe, Ni, Cu, Na, V, and Co in both species as well as Si in oilseed rape and Mg in wheat. It must also be pointed out that some nutrients belonging to this category were highly sequestered in roots such as Co (91 and 98% in roots for oilseed rape and wheat, respectively) and Na in wheat (93% vs. only 51% in oilseed rape). The second category are nutrients for which the relative root amount reflected their relative contribution to total biomass, such as N, P, S, K, Mn, Al, and Se in both species as well as Mg in oilseed rape. The last category included nutrients with a low root accumulation (Ca, B, and Cl in oilseed rape and Mo and Si in wheat).

For a given macronutrient deprivation (Figure 2), relative root amounts of 20 mineral nutrients (including the deprived nutrient) were monitored in both species (Figures 2A,B). Overall, the 6 macronutrient deprivations led to (i) a significant decrease (*p* < 0.01) in 19 relative root nutrient amounts in oilseed rape and 14 in wheat and (ii) a significant increase in the relative root nutrient amount of 16 and 30 nutrients in oilseed rape and wheat, respectively. For example, in both species, in Ca- and Mg-deprived plants, the strong decrease in the relative root amounts of Ca and Mg suggests that these two nutrients were remobilized from the roots to the shoots (Figures 2A,B). Similarly, in both species the relative root amount of Na was also significantly decreased in response to all macronutrient deprivations (except under -Mg). In contrast, in both species, the increase in relative V amount in S-deprived plants indicated that the lack of S availability led to an accumulation of V in the roots of both species. It can be noted that while some nutrients moved in the same direction in both plants (e.g., Na) in response to a given nutrient deprivation, other nutrients moved in opposite directions in the roots of the two species. For example, N deprivation resulted in a decrease in the relative amount of Mn in the roots of rapeseed and an increase in wheat roots.

### Plant Mineral Nutrient Net Uptake and Relative Tissue Contents Under Deprivation

**FIGURE 3:**
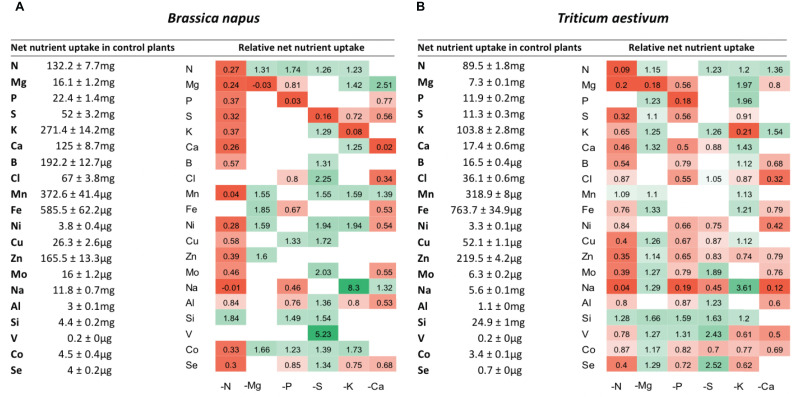
Net uptake of mineral nutrients by control plants over 10 days (left side) and heatmaps (right side) of relative mineral nutrient uptake by *B. napus* **(A)** and *T. aestivum* **(B)** plants after 10 days of macronutrient deprivation under hydroponic conditions. Relative mineral nutrient uptake was calculated (see section “Materials and Methods”) as the ratio of nutrient taken up by deprived plants/nutrient taken up by control plants. Only values significant for *p* < 0.01 are given.

**FIGURE 4:**
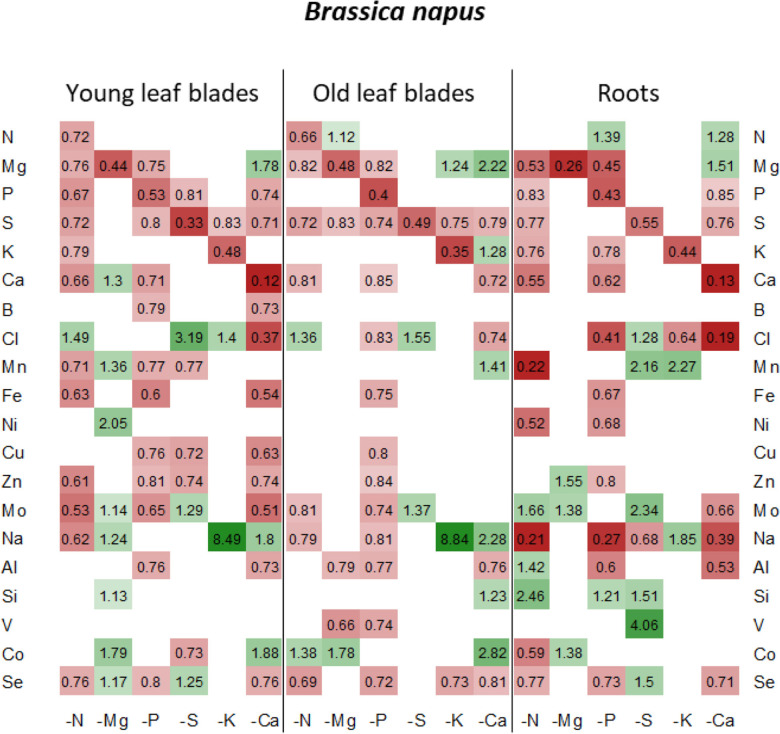
Heatmap of relative mineral nutrient concentrations in *B. napus* plants after 10 days of macronutrient deprivation under hydroponic conditions. Relative mineral nutrient concentration was calculated (see section “Materials and Methods”) as the ratio of nutrient concentration in deprived plants/nutrient concentration in control plants. Tissues developed before or after D_0_ are indicated as “young” or “old” as follows: young leaf blades, old leaf blades, young petioles and old petioles. Only values significant for *p* < 0.05 are given.

**FIGURE 5:**
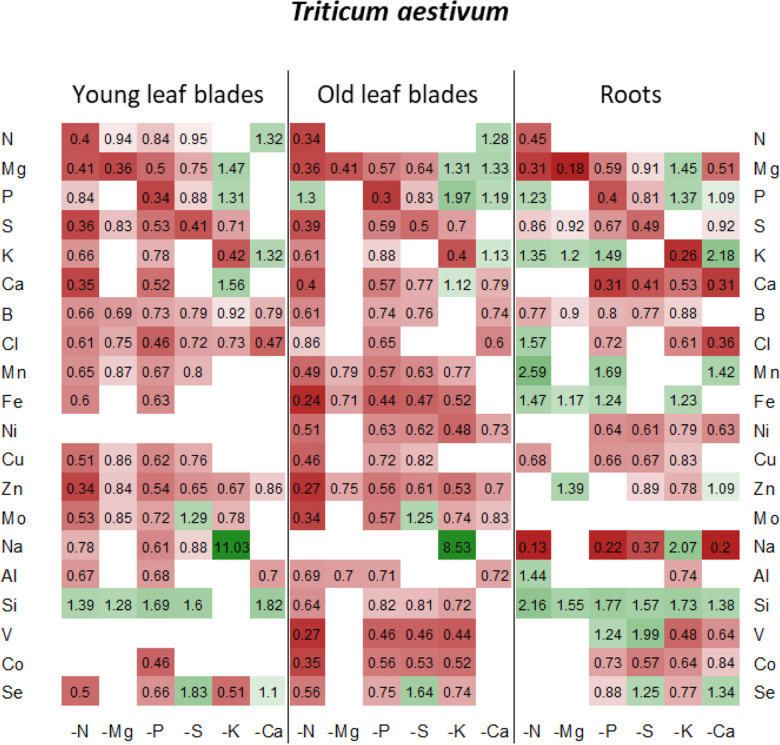
Heatmap of relative mineral nutrient concentrations in *T. aestivum* plants after ten days of macronutrient deprivation under hydroponic conditions. Relative mineral nutrient concentration was calculated (see section “Materials and Methods”) as the ratio of nutrient concentration in deprived plants/nutrient concentration in control plants. Tissues developed before or after D_0_ are indicated as “young” or “old” as follows: young leaf blades, old leaf blades, young petioles and old petioles. Only values significant for *p* < 0.05 are given.

The net uptake of each nutrient was calculated as the net difference in the concentration of each nutrient between Day 0 (beginning of nutrient deprivation) and Day 10 of macronutrient deprivation. The net uptake of macronutrients (N, Mg, P, S, K, and Ca), micronutrients (B, Cl, Mn, Fe, Ni, Cu, Zn, Mo) and beneficial elements (Na, Si, Al, V, Co, and Se) in control plants and their relative net uptake in deprived plants (−N, −Mg, −P, −S, −K, and −Ca) are presented in Figure 3. As expected, the net uptake of the deprived nutrients significantly decreased in most of the corresponding nutrient deprivation conditions, reaching less than about 10% of the net uptake in control plants. Indeed, significant reductions in the relative contents of these deprived nutrients were observed in all tissues, regardless of the plant species (Figures 4,5), except for N in oilseed rape roots (Figure 4).

Under each of the macronutrient deficiencies, two thirds of the 66 significant variations in the net uptake of the other nutrients (excluding the deprived nutrient of each deficiency) observed in oilseed rape showed similar trends to the 93 variations detected in wheat (Figure 3). Among them, N deficiency led to a significant decrease in most of the net nutrient uptakes for both species except for Si (1.84 ± 0.05 and 1.28 ± 0.03-fold increases in oilseed rape and wheat, respectively). Similarly, the relative net uptake of Si significantly increased under -P and -S in oilseed rape (Figure 3A) and under Mg, -P, -S, and -K in wheat plants (Figure 3B). This increase in net Si uptake was associated with a significant increase in Si concentration in the roots of both species (Figures 4, 5) and in the YLBs of wheat (Figure 5).

In oilseed rape (Figure 3A), Na and Mn were the most affected elements under N deprivation, with surprisingly an almost total cessation of their net uptake (0.04 ± 0.06 and −0.01 ± 0.02 for Mn and Na relative uptake, respectively) while Na was also strongly affected in wheat (Figure 3B, 0.04 ± 0.02). These interactions between N, Na, and Mn will be discussed later on. In Mg-deprived plants, the relative net uptakes of N, Mn, Fe, Zn, and Co in both species and of most other nutrients (P, S, K, Ca, Cu, Mo, Na, Si, V, and Se) in wheat (Figure 3B) were significantly increased. However, although the relative contents of these nutrients were higher in most Mg-deprived oilseed rape tissues (Figure 4), in wheat they only increased in the roots under –Mg conditions (Figure 5). It is noteworthy that none of the relative net nutrient uptakes decreased significantly in response to Mg deprivation (apart from Mg) (Figures 3A,B).

Under P deficiency, the relative net uptakes of Cl, Al, Se, and especially Na were strongly reduced in both species, whereas a larger range of nutrients had reduced net uptakes (S, Ca and most micronutrients) in wheat (Figure 3B). Si was the only nutrient with a higher net uptake in both species, while the N and V relative net uptakes were also increased in oilseed rape and wheat, respectively. The relative net uptakes of Co in the two species showed opposite trends, increasing in oilseed rape but decreasing in wheat.

**FIGURE 6:**
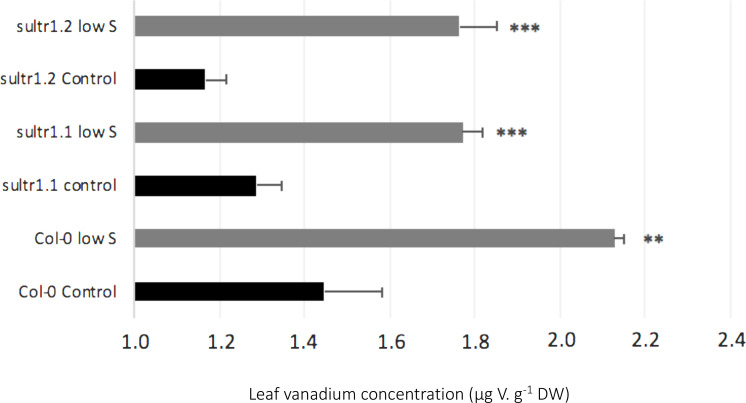
Effects of S deficiency on vanadium concentration in leaves of *Arabidopsis thaliana* plants, wild type Col-0 and the mutants AtSultr1.1 (KO) and AtSultr2.1 (point mutated, Shibagaki et al., 2002), grown under sufficient S supply (Control) or under low S. Data are given as the mean ± SE (*n* = 4) and are significantly different between control and low S at ^∗∗^*P* < 0.01) or ^∗∗∗^*P* < 0.001.

In S-deprived plants, positive interactions were associated with N, K, Cl, Mo, Al, Si, V, and Se in both species (Figure 3) and B, Mn, Ni, Cu, Co in oilseed rape (Figure 3A). These increased uptakes in oilseed rape resulted in a significant increase in Cl concentrations in leaf blades (young and old) and roots of oilseed rape (Figure 4). However, see section “Materials and Methods” these increases in Cl uptake (and concentrations) could be partially explained by the slightly higher Cl concentration in the S- and N-deprived nutrient solutions than in the control (2.7 mM and 4.6 mM vs. 2 mM, Supplementary Data SD1). Increases in V, Mo and Se net uptakes in S-deprived plants in both species (by a factor of 5.23 ± 0.32, 2.03 ± 0.17, and 1.34 ± 0.11, respectively, in oilseed rape and by 2.43 ± 0.11, 1.89 ± 0.06, 1.89 ± 0.06, and 2.52 ± 0.08, respectively, in wheat) (Figures 3A,B) were associated with an increase in Mo in all oilseed rape tissues (Figure 4) and wheat leaves (Figure 5) and an increase in V concentrations in the roots of both species (Figures 4, 5). The analysis of leaf V concentration in *Arabidopsis sultr1.1 KO* mutants *and sultr1.2 sel1-8* mutants showed that their capacity for root V uptake was increased under S deficiency, but not as much as in the wild type (Figure 6). This suggests that both SO_4_^2–^ transporters were able to mediate the uptake of VO_4_^2–^ as they do for other compounds with similar structural properties like MoO_4_^2–^, SeO_4_^2–^, and of course SO_4_^2–^. Similar to P deprivation, relative net Co uptake showed opposite trends in the two species, increasing in oilseed rape but decreasing in wheat.

In K-deprived oilseed rape and wheat plants (Figures 4, 5), the relative net uptake of N, Mg, Ca and Mn was increased. In the same way, net Na uptake was strongly increased (by a factor of 8.3 ± 0.03 and 3.61 ± 0.01 for oilseed rape and wheat, respectively) and led to a higher Na concentration in the leaf blades of both species (Figures 4, 5). Other positive interactions that were more specific to the plant species included Ni and Co in oilseed rape and P, B, Fe, Cu, and Si in wheat. The relative net uptakes of S and Se decreased in both species while the relative net uptakes of Al in oilseed rape (Figure 3A) and Cl, Zn, V, and Co in wheat (Figure 3B) decreased under K deprivation.

Finally, in the Ca-deprived plants of both oilseed rape and wheat, a negative effect on the relative net uptake of a large range of nutrients was observed, including Cl, Fe, Ni, Mo, Al in both species, P, S, Se in oilseed rape (Figure 3A), and Mg, B, Zn Na, V, and Co in wheat (Figure 3B). For example, the decrease in Cl uptake led to a very low Cl concentration in the YLBs and roots of both species in particular (Figures 4, 5). In contrast, only the relative net uptakes of N and K in wheat and Mn, Mg and Na in oilseed rape were significantly increased, and in the case of the two latter nutrients oilseed rape and wheat showed opposite trends in their uptake responses under Ca deprivation.

## Discussion

Previous work (Maillard et al., 2015, Maillard et al., 2016a; Courbet et al., 2019) has suggested that analysis of the plant functional ionome can be used to identify the physiological and molecular interactions that occur during mineral deprivations. In this study, changes in the functional ionome during macronutrient deficiencies have been observed in oilseed rape and wheat before any significant change in plant growth was recorded (Figure 1). Thus, under such conditions, the net uptake of each nutrient can be quantified and compared between deprivations of each of the 6 macronutrients without significant interference to plant growth.

First of all, nearly all mineral nutrient concentrations were higher in oilseed rape than wheat, regardless of the plant tissue examined (Table 1). For example, in control plants, the concentration of B, an element that is known to play a key role in cell wall structure, was about 10-fold higher in mature oilseed rape leaves than in wheat (Table 1). The opposite conclusion can be drawn for Si content, which was about 38-fold higher in mature wheat leaves than in oilseed rape (Table 1). Si is considered to be a beneficial element, probably taken up by aquaporin family and notably by Lsi1 (Mitani et al., 2008; Ma et al., 2011). Ma et al. (2011) showed that Lsi1 was involved in Si uptake from the soil in the form of silicic acid, in roots of monocotyledon and dicotyledon, then Si was transported in aerial part of plant by Lsi2, an active efflux transporter. Significant increases in grain yield and dry matter were observed when two wheat genotypes (Auqab-2000 and SARC-5) subjected to NaCl received supplementary Si (Tahir et al., 2006). Moreover, in poaceous species (such as wheat), which are considered Si-accumulating species (Marschner(eds), 2012), this element is known to reinforce the cell wall specifically when plants experience biotic and abiotic stresses (Epstein, 1999). Another difference between the two plant species involves the partitioning of Si between plant tissues. Indeed, about 10% of the relative Si content is localized in the roots of wheat (Figure 2B) compared to about 40% in oilseed rape (Figure 2A), irrespective of the nature of the nutrient deprivation, thus indicating that a higher proportion of the Si taken up by roots was translocated to the shoots in wheat. On the other hand, the relative root nutrient amounts (Figure 2) revealed that some nutrients were more immobile in wheat roots (well above 40%, such as Mg, Fe, Ni, Cu, Zn) than in oilseed rape roots (mostly below 30%), for all deprivations. Nutrient transport from roots to shoots is known to be regulated by the Casparian barrier, which blocks apoplastic flow in roots (Barberon et al., 2016). Furthermore, Ma et al. (2011) and Olsen and Palmgren (2014) suggested that monocotyledonous plants (such as cereals) possess an additional selective Casparian barrier, localized between the cortex and endodermis. According to this assumption it is possible that the second Casparian barrier might contribute to this wheat-specific sequestration of nutrients in the roots. Overall, these results clearly indicate that the ionome is specific to the plant tissue in question, as well as the plant species, even though the ionome shows a large degree of plasticity under different types of macronutrient deprivation.

One of the first processes that may affect the plant ionomic composition under macronutrient deprivation is remobilization of endogenous macronutrients from the source organs to sink organs. Under our experimental conditions, net remobilization of the deprived nutrient can be assessed. For example, the strong decrease in Mg and Ca in roots of both species suggests that these elements are massively remobilized from the roots to the shoots. On the other hand, the fact that the concentration in old leaves of wheat and oilseed rape (Figures 4, 5) dropped during deprivation to a greater degree than in young leaves suggests that remobilization of N, P, S, and K occurred in both species. This remobilization of macronutrients toward growing tissues may explain why plant growth was maintained for at least 10 days despite the macronutrient deprivation.

Regarding the effects of macronutrient deprivations on other elements, this study revealed 48 analogous patterns of change in relative net nutrient uptake between oilseed rape and wheat (Figure 3). Amongst these effects, negative interactions such as the impact of N deficiency on plant metabolism *via* protein synthesis and alteration of resource allocation are already well known (Crawford, 1995; Scheible et al., 2004). In this study, N starvation induced a decrease in the uptake of almost all mineral nutrients in both species, except for Si. According to other work conducted on rice, this exception for Si could be due to an antagonistic interaction between N and Si during growth enhancement in this species (considered a Si-accumulator) (Wu et al., 2017). Indeed, Wu et al. (2017) demonstrated that an increase in N fertilization reduced leaf Si content significantly, and in contrast, Si supply downregulated gene expression (*amt1.1* and *nrt1.1*) involved in N uptake in wheat. Contrastingly, recent results obtained in oilseed rape (a non Si-accumulator species) have indicated that a Si root supply upregulates the gene encoding a nitrate transporter (*BnaNRT2.1)* and enhances N uptake (Haddad et al., 2018). Overall, our results also suggest that Si, which is considered a beneficial element, could interact with N, one of the major macronutrients for plant growth and yield.

In a very similar way to N deprivation, P deficiency revealed numerous elemental interactions. Indeed, the net uptakes of Mg, Cl, Na, Al, and Se decreased in oilseed rape and wheat, as did the uptakes of S, Ca, B, Ni, Cu, Zn, Mo, and Co in wheat and Fe in oilseed rape. This could be explained by the major role that P plays in plant metabolism because this macronutrient is central to the structure of adenosine triphosphate (ATP) and is responsible for its high-energy proprieties (Neupane et al., 2019). Plaxton and Tran (2011) explained that under P deprivation, Pi-use efficiency is increased by upregulation of Pi-starvation-inducible hydrolases that trap and recycle P *via* extra and intracellular organic P to maintain plant metabolism. Nevertheless, P deficiency cannot be continuously offset, so this means that any ATP decrease will restrict plant photosynthesis function due to Rubisco deactivation (Butz and Sharkey, 1989; de Groot, 2003) and also the uptake of some nutrients that are usually ATP-dependent processes in the root.

Our results also provided evidence about novel negative interactions between N, P and Na in both species. These results suggest that Na transport could be functionally related to N uptake and this may be illustrated by data extracted from the Ionomic Hub (Salt et al., 2008), which contains an ionomic analysis of *Arabidospis* nitrate transporter mutant lines. For example, compared to the parent line, there were significant reductions of 39 and 48% (*p* ≤ 0.001) in the leaf Na concentration in an *nrt2.1 Arabidopsis thaliana* mutant (SALK_035429 homozygote knock-out line) cultivated on 2 different soils. It is usually assumed that NRT2.1, a high-affinity nitrate transporter, works as a NO_3_^–^/2H^+^ symporter that requires energy for H^+^/ATPase activity. Consequently, the secondary efflux of H^+^ probably coupled with a Na^+^ influx could partially explain the strong decrease in Na uptake shown in N-deprived plants. On the contrary, compared with the parent line, the *nrt1.1* (SALK_097431 homozygote knock-out line) mutant showed similar or increased Na concentration in leaves compared to the parent line when cultivated on the two different soils. This increase in leaf Na concentrations in the *nrt1.1* KO mutant could be the consequence of a compensatory increase in NRT2.1 expression, as previously reported by Munos et al. (2004), and could explain the increased H^+^/Na^+^ antiporter activity that in turn increased Na^+^ uptake. Alvarez-Aragon and Rodríguez-Navarro. (2017) suggested that under saline conditions two nitrate-dependent transport systems that work in series to take up and load Na^+^ into the xylem constitute the major pathway for the accumulation of Na^+^ in *Arabidopsis thaliana* shoots. They also found that Na^+^ accumulation in the *nrt1.1* mutant was partially defective, suggesting that NRT1.1 either partially mediates or modulates nitrate-dependent Na^+^ transport.

In the same way, relative net uptake of Na^+^ was also strongly decreased in P-deprived plants (Figures 3A,B) and led to a reduction in the relative amount (Figures 2B,C) and concentration of Na in roots (Figures 4, 5). Previous work has indicated that the uptake of Pi is mediated by different PHO1 and PHT transporters that require energy from H^+^/ATPase activity (Hamburger et al., 2002; Briat et al., 2015). From our results and data from the literature, it could be hypothesized that these transport systems are also connected to a Na^+^/H^+^ antiporter that is inefficient when Pi is not available, thus leading to a decrease in Na^+^ uptake (Shi et al., 2002; Saito and Uozumi, 2020).

Ca deprivation also negatively affected the net uptake of several mineral nutrients in oilseed rape and wheat. This result is very interesting because to our knowledge no nutrient interactions have been previously reported in Ca-deprived plants. At the cellular level, Ca plays the role of second messenger in the regulation of major metabolic and physiological processes such as photomorphogenesis, drought resistance, cold adaptation and thermotolerance (Anil and Rao, 2001). Furthermore, Ca is essential for the structure of cell walls because it is bound to pectate during the strengthening of plant tissues (Olle and Bender, 2009).

It is notable that the macronutrient deprivation experiments highlighted 20 positive interactions common to both species, some of them already described, such as a strong increase in Na uptake under K deprivation (Figure 3) coupled with a reduction in its relative content in roots (Figure 2) and a massive accumulation in leaves (Figures 4, 5). K is one of the most abundant cations in plants (Mäser et al., 2002), principally known for its osmotic role, and it is regulated via the KUP potassium transporter family, which contributes to K^+^ uptake under K starvation or under drought stress (Osakabe et al., 2013; Nieves-Cordones et al., 2016). Most HKT transporters are Na^+^ transporters, but a few are regarded as Na^+^/K^+^ symporters (Wang and Wu, 2013). Mäser et al. (2002) suggested multiple pathways for root Na^+^ uptake such as permeation of Na^+^ through K^+^ and Ca^2+^ transporters, use of Na^+^ transport to energize K^+^ uptake, and also Na^+^-selective uptake. Sodium is a beneficial element whose function is not well understood (Alvarez-Aragon and Rodríguez-Navarro, 2017) but our data show a potential substitution of K^+^ by Na^+^ under our K^+^-deprivation conditions, suggesting at least a transient osmotic compensation.

Under S deprivation there was surprisingly a greater effect on mineral nutrient uptake in wheat than in oilseed rape (Figure 3). Nevertheless, novel positive interactions between S, Mo, V and Se could be identified in both species. As detailed in the introduction, S as a macronutrient is involved in multiple interactions with other components of the functional ionome (Courbet et al., 2019) such as the synthesis of Mo-cofactor, which also requires Fe, Zn and Cu, as well as the synthesis of Fe-S clusters. Moreover, it has been shown that the strong over-expression of sulfate transporters triggered by S deficiency (Maruyama-Nakashita et al., 2004) leads to an increased uptake of Mo and Se in the form of MoO_4_^2–^ and SeO_4_^2–^, respectively (Shinmachi et al., 2010; Maillard et al., 2016a), as also found in this study (Figure 3). Moreover, we observed that under S deprivation, V uptake is 6 and 2 times higher in oilseed rape and wheat, respectively, compared to control plants (Figure 3). These results suggested that V could be taken up by sulfate transporters, and probably in the form of vanadate (VO_4_^3–^), which is also supported by the analysis of leaf V concentration in *Arabidopsis sultr1.1* KO mutants *and sultr1.2* (*sel1-8*) mutants subjected to S deficiency (Figure 6). This suggests that both transporters are able to mediate the uptake of VO_4_^2–^ as they do for other compounds with similar structural properties (SO_4_^2–^, MoO_4_^2–^, SeO_4_^2–^). The use in this study of *Arabidopsis sultr1.1 KO* mutants and *sultr1.2 (sel1-8)* mutants for V analysis was derived from samples of a previous study that has been previously published (Maillard et al., 2016a) in which the expression of genes encoding sulfate transporters was already given for the wild type and the mutants. Lastly, it must be pointed out that the stimulation of V uptake found in this study under S deprivation occurred in the absence of any added V, with the element only being present at an ultra-trace level in the nutrient solution (<0.01 ppm), thus suggesting a high affinity of SULT transporters for VO_4_^2–^.

## Conclusion

In conclusion, this work demonstrates that the ionomic composition is tissue and species specific. While usually considered as being tightly regulated, our work shows the true plasticity of the ionome composition when plants are exposed to macronutrient deprivation. The concentrations of the deprived nutrients sharply declined, allowing the plants to maintain a similar growth rate for at least 10 days, and this is probably the result of remobilization toward actively growing tissues such as young leaves. A large number of positive and negative interactions between nutrients were demonstrated, some of them being common to the two species studied. Numerous mechanisms and crosstalks, which will require further work, may be involved in direct (S, Se, V, Mo) or indirect interactions (NO_3_^–^ and Na^+^) at the level of root transporters, while a substitution mechanism may be hypothesized between a number of the cations (Ca and Mg, K and Na). The consequences of micronutrient and beneficial nutrient deprivations will be presented in a companion paper (D’Oria et al., 2020), together with an analysis of the specificity of ionomic signatures.

## Data Availability Statement

The original contributions presented in the study are included in the article/Supplementary Material, further inquiries can be directed to the corresponding author/s.

## Author Contributions

All authors listed have made a substantial, direct and intellectual contribution to the work, and approved it for publication.

## Conflict of Interest

The authors declare that the research was conducted in the absence of any commercial or financial relationships that could be construed as a potential conflict of interest.

## Supplementary Material

Supplementary Data SD1 Averaged nutrient concentrations (ppm) of *B. napus* leaf (young and old leaves together) grown in field conditions [from Sarda et al. (2013) and Maillard et al. (2016b) corresponding to a maximum of 194 agricultural plots randomly harvested in France], or grown under hydroponic conditions [samples from Maillard et al. (2016a) or from this paper and the companion paper D’Oria et al. (2020)] with different nutrient solutions. Leaf nutrient concentrations obtained under hydroponic cultures, were averaged from all treatments (control plants and plants submitted to macro, micro or beneficial nutrients deficiencies) to maximize the range of concentrations encountered in hydroponic conditions compare to field experiments. Values are given as the mean ± SD for the corresponding number of leaf samples (n). For each nutrient concentration, a ratio between hydroponically [from Maillard et al. (2016b), ratio 1; from this study and companion paper D’Oria et al. (2020), ratio 2] and field grown plants is also given.

Supplementary Data SD2 Compositions of the nutrient solutions modified from Hoagland solution according to Maillard et al. (2016a, b) to best match the hydroponic ionome to field observations (data not shown). These solutions were used to expose *Brassica napus L*. and *Triticum aestivum* to 10 days of single-element deprivations of nitrogen (N), magnesium (Mg), phosphorus (P), sulfur (S), potassium (K), or calcium (Ca).

## Abbreviations

OLB: old leaf blades
OP: old petioles
YLB: young leaf blades
YP: young petioles.
