# Supplementary material for: Specificity and Plasticity of the Functional Ionome of Brassica napus and Triticum aestivum Subjected to Macronutrient Deprivation
Source: Front Plant Sci. 2021 Feb 2;12:641648. doi: 10.3389/fpls.2021.641648 (PMC7891181; doi:10.3389/fpls.2021.641648)
Supplement: Supplementary Data SD1 — Averaged nutrient concentrations (ppm) of B. napus leaf (young and old leaves together) grown in field conditions [from Sarda et al. (2013) and Maillard et al. (2016b) corresponding to a maximum of 194 agricultural plots randomly harvested in France], or grown under hydroponic conditions [samples from Maillard et al. (2016a) or from this paper and the companion paper D’Oria et al. (2020)] with different nutrient solutions. Leaf nutrient concentrations obtained under hydroponic cultures, were averaged from all treatments (control plants and plants submitted to macro, micro or beneficial nutrients deficiencies) to maximize the range of concentrations encountered in hydroponic conditions compare to field experiments. Values are given as the mean ± SD for the corresponding number of leaf samples (n). For each nutrient concentration, a ratio between hydroponically [from Maillard et al. (2016b), ratio 1; from this study and companion paper D’Oria et al. (2020), ratio 2] and field grown plants is also given. [file Data_Sheet_1.PDF]

	Field grown plants From Sarda *et al.* (2013) and Maillard *et al.* (2016b)			Hydroponically grown plants from Maillard *et al.* (2016a)			Hydroponically grown plants from this study, and companion paper D'Oria *et al.* (2020)				
	**mean**	**sd**	**n=**	**mean**	**sd**	**n=**	**mean**	**sd**	**n=**	**ratio 1**	**ratio 2**
**N**	24 059	± 8804	151	42 286	± 13315	166	43 374	± 10953	200	1.76	1.80
**Mg**	1 274	± 580	194	3 761	± 2196	166	4 237	± 2407	200	2.95	3.33
**P**	2 830	± 978	194	4 354	± 2350	166	5 426	± 2172	200	1.54	1.92
**S**	6 228	± 3755	194	10 944	± 5403	166	14 895	± 5723	200	1.76	2.39
**K**	23 195	± 9042	194	30 797	± 10975	166	44 996	± 12391	200	1.33	1.94
**Ca**	33 569	± 19496	194	42 432	± 26961	166	34 792	± 20955	200	1.26	1.04
**B**	29	± 18	194	44	± 20	166	69	± 27	200	1.50	2.37
**Cl**	7 298	± 3520	41	10 301	± 5598	35	12 741	± 6162	200	1.41	1.75
**Mn**	64	± 44	194	186	± 104	166	150	± 77	200	2.91	2.36
**Fe**	652	± 708	192	103	± 46	166	121	± 42	200	0.16	0.19
**Ni**	1.4	± 1.6	191	0.26	± 0.11	30	0.9	± 1.16	200	0.18	0.62
**Cu**	3.7	± 1.8	194	4.0	± 3.1	166	6.9	± 6.1	200	1.09	1.87
**Zn**	29	± 22	188	40	± 30	161	49	± 25	200	1.39	1.67
**Mo**	2.1	± 1.8	194	177	± 176	166	4.5	± 2.1	200	84.71	2.18
**Na**	1 055	± 1391	194	600	± 1240	166	1 391	± 1965	200	0.57	1.32
**Al**	2 121	± 1973	39	348	± 97	35	505	± 158	200	0.16	0.24
**Si**	1 323	± 1146	40	581	± 265	43	1 074	± 280	200	0.44	0.81
**Co**	0.33	± 0.34	192	0.11	± 0.06	31	0.25	± 0.29	200	0.33	0.76
**Se**	0.14	± 0.14	194	0.05	± 0.03	32	0.90	± 0.34	200	0.34	6.55
