# Supplementary material for: Specificity and Plasticity of the Functional Ionome of Brassica napus and Triticum aestivum Subjected to Macronutrient Deprivation
Source: Front Plant Sci. 2021 Feb 2;12:641648. doi: 10.3389/fpls.2021.641648 (PMC7891181; doi:10.3389/fpls.2021.641648)
Supplement: Supplementary Data SD2 — Compositions of the nutrient solutions modified from Hoagland solution according to Maillard et al. (2016a, b) to best match the hydroponic ionome to field observations (data not shown). These solutions were used to expose Brassica napus L. and Triticum aestivum to 10 days of single-element deprivations of nitrogen (N), magnesium (Mg), phosphorus (P), sulfur (S), potassium (K), or calcium (Ca). [file Data_Sheet_2.PDF]

Salts	Salt concentrations (µM) of deprived solutions :						
	Control	-N	-Mg	-P	-S	-K	-Ca
KNO_3	1000	0	1000	1000	1000	0	3500
$Ca(NO_3)_2 + 4H_2O$	1250	0	1250	1250	1250	1750	0
KH_2PO_4	200	0	200	0	0	0	0
K_2HPO_4	0	200	0	0	200	0	0
$MgSO_4$	400	150	0	400	0	400	400
$MgCl_2$	0	250	0	0	400	0	0
NaFe-EDTA $3H_2O$	50	50	50	50	50	50	50
NaFe-EDDHA	50	50	50	50	50	50	50
Fe-Citrate	0	0	0	0	0	0	0
H_3BO_3	10	10	10	10	10	10	10
$MnSO_4$	3	3	3	3	0	3	3
$MnCl_2$	0	0	0	0	3	0	0
$ZnSO_4$	3	3	3	3	0	3	3
$CuSO_4$	0.7	0.7	0.7	0.7	0	0.7	0.7
$(NH_4)_6Mo_7O_{24}$	0.008	0.008	0.008	0.008	0.008	0.008	0.008
$CoCl_2$	0.1	0.1	0.1	0.1	0.1	0.1	0.1
$NiCl_2$	0.15	0.15	0.15	0.15	0.15	0.15	0.15
$Si(OH)_4$	900	900	900	900	900	900	1100
$CaCl_2 + 2H_2O$	500	750	500	200	0	450	0
KCl	100	400	0	300	100	0	0
Na_2SeO_4	0.01	0.01	0.01	0.01	0.01	0.01	0.01
$CaCO_3$	360	940	0	620	720	0	0
K_2SO_4	100	350	150	100	0	0	100
Na_2SiO_3	200	200	200	200	200	200	0
HCl	600	2220	500	1400	1800	300	900
$CaSO_4$	0	0	350	0	0	100	0
$Ca(H_2PO_4)$	0	0	0	0	0	200	0
$ZnCl_2$	0	0	0	0	3	0	0
$CuCl_2$	0	0	0	0	0.7	0	0
NaCl	0	0	0	0	0	0	200
NaH_2PO_4	0	0	0	0	0	0	200
KOH	0	0	0	0	0	0	1000

Nutrients	Elemental concentrations (µM) of deprived solutions :						
	Control	-N	-Mg	-P	-S	-K	-Ca
N	3500	0.048	3500	3500	3500	3500	3500
Mg	400	400	0	400	400	400	400
P	200	200	200	0	200	200	200
S	507	507	507	507	0	507	507
K	1500	1500	1500	1500	1500	0	4700
Ca	2110	1690	2100	2070	1970	2500	0
B	10	10	10	10	10	10	10
Cl	2001	4621	1701	2101	2707	1201	1101
Mn	3	3	3	3	3	3	3
Fe	100	100	100	100	100	100	100
Ni	0.15	0.15	0.15	0.15	0.15	0.15	0.15
Cu	0.7	0.7	0.7	0.7	0.7	0.7	0.7
Zn	3	3	3	3	3	3	3
Mo	0.056	0.056	0.056	0.056	0.056	0.056	0.056
Na	500	500	500	500	500	500	500
Al	0	0	0	0	0	0	0
Si	1100	1100	1100	1100	1100	1100	1100
V	0	0	0	0	0	0	0
Co	0.1	0.1	0.1	0.1	0.1	0.1	0.1
Se	0.01	0.01	0.01	0.01	0.01	0.01	0.01
